# Uterine Artery Embolization for Symptomatic Adenomyosis: 7-Year Clinical Follow-up Using UFS-Qol Questionnaire

**DOI:** 10.1007/s00270-017-1686-1

**Published:** 2017-05-17

**Authors:** Annefleur M. de Bruijn, Marieke Smink, Wouter J. K. Hehenkamp, Robbert J. Nijenhuis, Albert J. Smeets, Focco Boekkooi, Paul J. H. M. Reuwer, Willem J. Van Rooij, Paul N. M. Lohle

**Affiliations:** 1 Department of Gynecology, VU Medical Centre, De Boelelaan 1117, 1007MB Amsterdam, The Netherlands; 2 Department of Gynecology, Elisabeth Tweesteden Ziekenhuis, Tilburg, The Netherlands; 3 Department of Radiology, Elkerliek Ziekenhuis, Helmond, The Netherlands; 4 Department of Radiology, Elisabeth Tweesteden Ziekenhuis, Tilburg, The Netherlands

**Keywords:** Uterine artery embolization, Adenomyosis, UFS-QOL

## Abstract

**Purpose:**

The purpose of this study was to assess clinical outcomes 7 years after uterine artery embolization (UAE) in the treatment of symptomatic adenomyosis.

**Materials and Methods:**

In this prospective cohort study, one specialized hospital in the Netherlands recruited patients with symptomatic adenomyosis or adenomyosis in combination with fibroids for UAE. The 7-year post-intervention outcomes were health-related quality of life (HRQOL), symptom severity scores (SSS), satisfaction, menopause and re-interventions.

**Results:**

Twenty-nine patients with adenomyosis (15 with fibroids) were treated with UAE between September 2006 and January 2010. The 7-year questionnaire was mailed in November 2016. The mean follow-up was 95 months (SD 9.0) at a mean age of 50 (SD 5.4). Questionnaires were returned by 24/29 patients (83%). The remaining five patients were contacted through telephone. One of these patients was untraceable. Seven years after treatment 5 of 28 patients (18%) underwent a secondary hysterectomy. The HRQOL and SSS scores as measured by UFS-QOL at 3 months after UAE showed significant improvement of −57 points (score: 15) and +40 points (score: 91), respectively. These scores remained comparable stable up unto 7 years. The SSS showed a significant difference of 17 points (0–100) in favor of the adenomyosis in combination with fibroids group (*p* = 0.020). Menopause was reported by 10/28 patients (36%). Twenty-one of 29 (72%) patients declared to be at least fairly satisfied about UAE.

**Conclusions:**

After 7 years of follow-up, in 82% of UAE-treated patients with symptomatic adenomyosis a hysterectomy was avoided.

## Introduction

Adenomyosis is a benign disease characterized by the presence of ectopic endometrial glands and stroma which causes reactive hypertrophy of the myometrium [1, 2]. Uterine artery embolization (UAE) was first described in 1995 for the treatment of uterine fibroids [3]. It has been established as a valuable treatment option for patients with symptomatic uterine fibroids [4–6]. Since then uterine artery embolization is being explored as a possible treatment option for adenomyosis and seems to have a favorable outcome in multiple case series, although randomized controlled trials are lacking [7–14].

Earlier we reported the result of uterine artery embolization in the treatment of symptomatic therapy-resistant adenomyosis with 3-year follow-up. In that study, we analyzed clinical outcomes, health-related quality of life (HRQOL), symptom severity scores (SSS), menopause and satisfaction [15]. This 37-month follow-up reported preservation of the uterus in 28/29 patients (97%) with good clinical outcome. It is important to extent the follow-up period in order to further expand upon these outcomes. The purpose of this study was to assess clinical outcomes 7 years after uterine artery embolization in the treatment of symptomatic adenomyosis in 29 patients with pure adenomyosis (*n* = 14) or adenomyosis with fibroids (*n* = 15).

## Materials and Methods

### Study Design

The detailed methods in terms of inclusion criteria, exclusion criteria, MRI criteria, UAE procedures and UFS-QOL description of this study have been reported earlier [15].

In short, this prospective cohort study was conducted in one hospital in the Netherlands. It evaluated 234 symptomatic patients (abnormal menstrual bleeding, pelvic pain, and bulk-related symptoms) who presented between 2006 and 2010. Patients with MRI confirmed (junctional zone >12 mm) pure adenomyosis or adenomyosis in combination with fibroids were asked to participate in the study.

This 7-year follow-up study was approved by the local ethics committee. All procedures performed were in accordance with the ethical standards of the institutional and/or national research committee and with the 1964 Helsinki Declaration and its later amendments or comparable ethical standards. Patients gave their informed consent and filled out the standardized questionnaire.

### Study Measures

During the 3-year follow-up, patients received three similar HRQOL and SSS questionnaires (UFS-QOL) at different points in time. The 7-year questionnaire evaluated re-intervention rates, menopause, patient satisfaction, SSS and HRQOL and was sent approximately 7 years after the last patients were treated. If patients did not respond to the questionnaires telephone contact was attempted, we inquired about participation, additional treatment and patient satisfaction.

#### HRQOL and SSS

The UFS-QOL questionnaire was used to evaluated HRQOL and SSS. A higher HRQOL score means a better quality of life. A lower SSS stands for improvement of symptoms. As described earlier, patients with a SSS <20 in combination with an total HRQOL score >80 were considered asymptomatic [15].

#### Menopause

We inquired whether the patient went through menopause (the absence of menstrual periods for at least 12 months). Patients could choose from the answers “yes,” “no” or “I don’t know.” The last was applicable to patients who underwent hysterectomy or displayed amenorrhea after uterine artery embolization.

#### Satisfaction

Patients were asked to indicate how satisfied they were with the received treatment on a 7-point Likert scale: “very satisfied,” “fairly satisfied,” “not satisfied, nor unsatisfied,” “fairly unsatisfied,” “unsatisfied” or “very unsatisfied.”

We also inquired whether patients would recommend the primary treatment to a friend and whether or not they would have chosen uterine artery embolization again if they would have the opportunity to do so.

### Statistical Analysis

We used SPSS statistical software (version 22) for analyses. Comparison of differences between groups was assessed with the Mann–Whitney *U* test. Longitudinal differences between 3 and 7 years of follow-up were evaluated using the Wilcoxon signed-rank test. Hysterectomy timing during 7 years of follow-up was examined with Kaplan Mayer survival analysis.

A probability <0.05 was considered statistically significant.

## Results

Baseline results and outcomes until 3 years of follow-up were reported previously [15]. Table 1 presents an overview of these outcomes. A total of 29 women were enrolled: 14 patients with pure adenomyosis and 15 patients with combined adenomyosis/fibroids. Median baseline HRQOL scores in the pure adenomyosis and adenomyosis combined with fibroids group were comparable (*p* = 0.076). Median baseline SSS was significantly higher (=worse) in the pure adenomyosis group compared to the adenomyosis with fibroids group (*p* = 0.036). At 3 and 37 months of follow-up, HRQOL and SSS were comparable.

**Table 1:** Clinical follow up at baseline, 3 months, 3 year and seven years

Patients with adenomyosis (*n* = 29)	Pure: *n* = 14 Combined: *n* = 15	Pure: *n* = 13 Combined: *n* = 15	Pure: *n* = 13 Combined: *n* = 15	Pure: *n* = 10 Combined: *n* = 11
	Baseline (*p* = 0.036)	3 months (*p* = 0.835)	3-year follow-up (*p* = 0.382)	7-year follow-up (*p* = 0.020)
SSS				
Pure adenomyosis (median)	78.2	18.8	15.6	17.2
Adenomyosis with fibroids (median)	62.4	15.6	6.3	0
Overall (median, range)	72 (23–100)	15 (0–66)	17 (0–34)	17.2 (0–43.8)

Patients with adenomyosis (*n* = 29)	Pure: *n* = 14 Combined: *n* = 15	Pure: *n* = 13 Combined: *n* = 15	Pure: *n* = 13 Combined: *n* = 15	Pure: *n* = 10 Combined: *n* = 11
	Baseline (*p* = 0.076)	3 months (*p* = 0.769)	3-year follow-up (*p* = 0.091)	7-year follow-up (*p* = 0.379)
HRQOL				
Pure adenomyosis (median)	37.9	91.4	89.7	95.3
Adenomyosis with fibroids (median, range)	53.0	87.1	99.1	99.1
Overall (median, range)	51 (20–88)	91 (55–100)	99 (29–100)	98.3 (9–100)

Patients with adenomyosis (*n* = 29)	Pure: *n* = 14 Combined: *n* = 15	Pure: *n* = 13 Combined: *n* = 15	Pure: *n* = 13 Combined: *n* = 15	Pure: *n* = 10 Combined: *n* = 11
Additional treatment				
Secondary embolization	NA	NA	3 (1× pure group, 2× combined group)	Status quo
Secondary hysterectomy	NA	NA	1 (pure group)	4 (2× pure group, 2× combined group)

Median SSS, HRQOL scores and additional treatment

NA, not applicable; pure, only adenomyosis; combined, adenomyosis with concurrent fibroids

### Patients

The mean follow-up duration was 7.4 years (SD 0.83) at an overall mean age of 50.5 (SD 5.5). The mean age of patients in the pure adenomyosis and the adenomyosis with fibroids group was 48.8 (SD 7.1) and 51 (SD 4.6), respectively. Questionnaires were returned in 24/29 patients (83%). This included three patients who underwent secondary hysterectomy and therefore could not fill out the complete questionnaire. Completed questionnaires were returned in 21/29 patients (72%). These 21 filled in questionnaires were divided into 10 questionnaires from patients in the pure adenomyosis group and 11 questionnaires in the adenomyosis with fibroids group. We contacted the five non-responders by telephone and inquired about participation, additional treatment received, recommendation to a friend and satisfaction. Two of the non-responders underwent a secondary hysterectomy due to persisting symptoms, two patients (pure adenomyosis *n* = 1 and adenomyosis with fibroids *n* = 1) did not have a recurrence of symptoms, but declined full questionnaire participation and one patient was untraceable. These patients did answer the satisfaction questions concerning UAE.

### Clinical Outcome, HRQOL and SSS at 37 Month Follow-Up, as Already Reported [15]

In the earlier reported 37-month follow-up, three patients underwent a second UAE at 6, 7, and 14 months and one patient received a secondary hysterectomy at 17 months following UAE. The second embolizations were carried out in one patient with pure adenomyosis and in two patients with adenomyosis and fibroids. The hysterectomy occurred in a patient with pure adenomyosis. Of 29 patients, 22 (76%) reported to be asymptomatic and seven patients reported to have persisting mild symptoms without additional therapy. Four of these seven patients had pure adenomyosis.

**Fig. 1:**
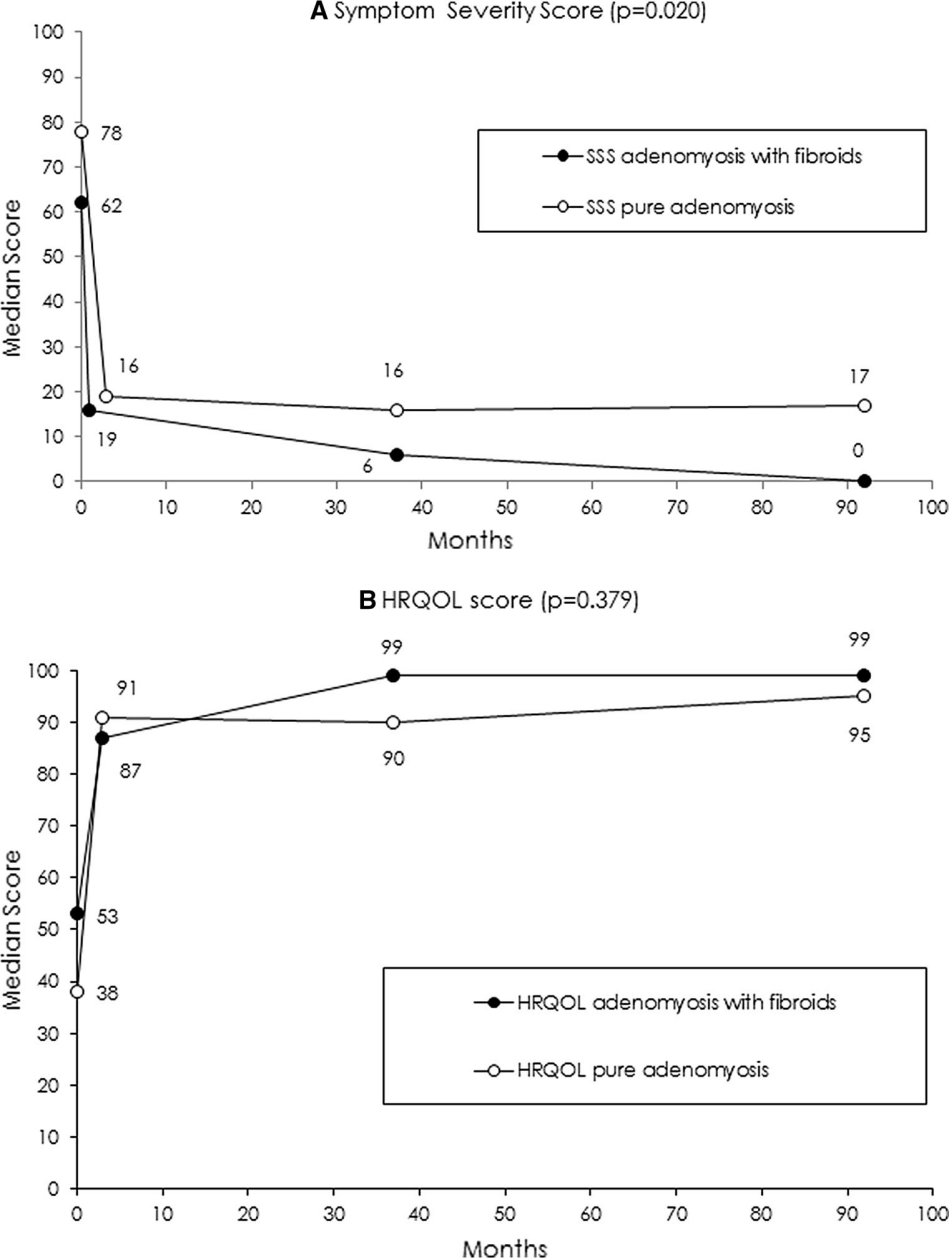
Median SSS and HRQOL at baseline, 3, 37 and 92 months. **A** Median SSS. **B** Median HRQOL. Both figures depict two groups: patients (*n* = 10) with pure adenomyosis and patients (*n* = 11) with adenomyosis and fibroids (excluding two patients who declined questionnaire participation, five patients who underwent hysterectomy and one patient who was untraceable)

Mean HRQOL scores are displayed in Table 1. Longitudinal analysis revealed improvement between baseline and 3-month follow-up (HRQOL: *p* < 0.001; Symptoms Severity: *p* < 0.001). During the 3–37-month interval, SSS improved further (*p* = 0.005) and HRQOL stabilized. Scores over time are displayed in Fig. 1.

### Clinical Outcome, HRQOL and SSS at 7 Years

**Fig. 2:**
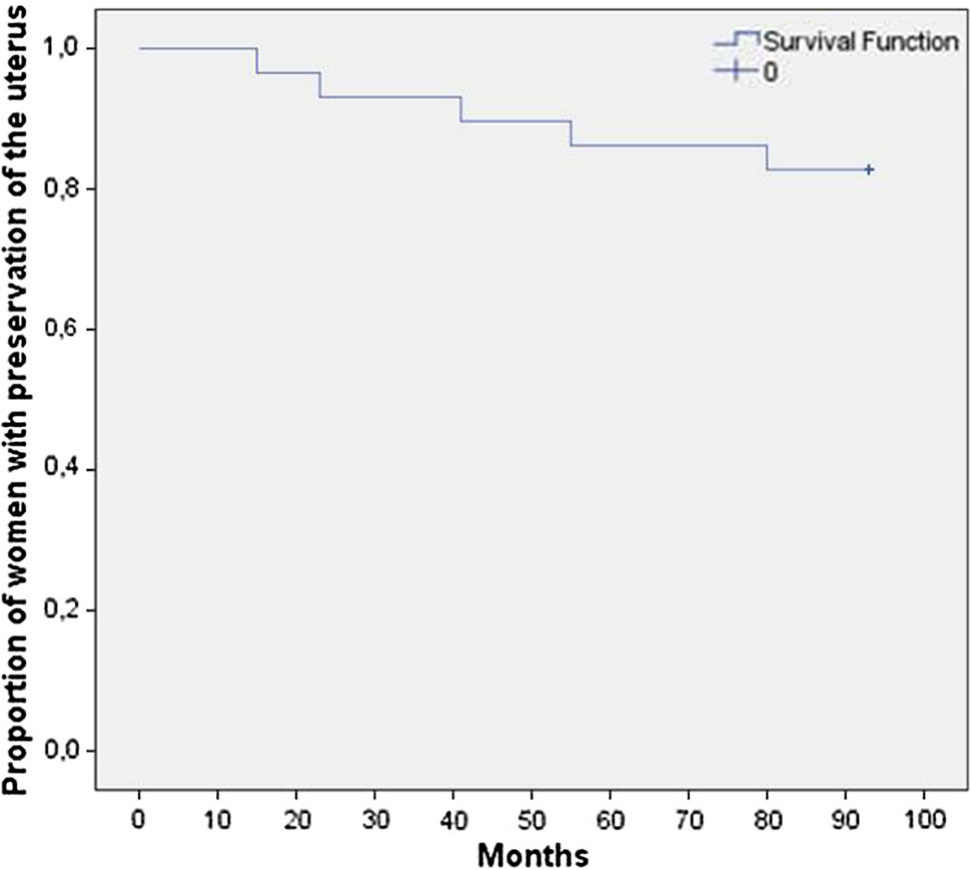
Secondary hysterectomy rate during long-term follow-up

At 7-year follow-up, four additional women had hysterectomy performed at 23, 41, 55 and 80 months after UAE. Thus, in total five of 28 women underwent a secondary hysterectomy (Fig. 2). Resulting in avoidance of hysterectomy in 23/28 (82%) of patients. Three of the hysterectomies occurred in the pure adenomyosis group (14, 23, 41 months) and two in the adenomyosis with fibroids group (55, 80 months). Seventeen of 23 (74%) patients with a preserved uterus were asymptomatic, four had persisting symptoms and two declined questionnaire participation. Persisting symptoms occurred in three patients with pure adenomyosis and in one patient with adenomyosis and fibroids.

Overall median SSS in 21 patients was 6.3 (range 0–87.5) corresponding with a median HRQOL overall score of 98.3 (range 8.6–100) (Table 1). SSS, HRQOL scores and secondary interventions for patients with pure adenomyosis and adenomyosis with fibroids are depicted in Table 1. Figure 1 shows median HRQOL and SSS over time for both groups Statistical analysis shows no HRQOL differences between groups at 7-year follow-up (*p* = 0.379). The SSS shows a statistical difference in favor of the adenomyosis with fibroids group (*p* = 0.020).

#### Menopause

At 7 years, 28/28 patients answered the following menopause question. The question: “did you experience absence of menstrual periods for at least 12 months?” was answered “yes” by 10/28 patients (36%). “No” was answered by 8/28 patients (28.6%) and 10/28 patients (36%) answered “I don’t know”. The last group consisted of 6/28 (21%) patients who reported permanent amenorrhea directly following UAE and four patients who underwent hysterectomy and therefore do not have periods. There was no difference between pure adenomyosis and adenomyosis with fibroids (*p* = 0.380). Table 2 reports menopause outcomes per group.

**Table 2:** Menopause outcomes per group

	Pure adenomyosis (*n* = 14)	Adenomyosis with fibroids (*n* = 14)	*p* value
Yes*	6 (42.9%)	4 (28.6%)	0.380
No*	4 (28.6%)	4 (28.6%)	
I don’t know*	4 (28.6%)	6 (42.9%)	

* Response to question “did you experience absence of menstrual periods for at least 12 months?”

#### Satisfaction

At 7 years, 24/29 patients responded to questions concerning treatment satisfaction, advising UAE to a fiend and undergoing UAE again. Within groups, 11 patients in the pure adenomyosis group and 13 patients in the adenomyosis with fibroids group responded to the following questions. In five patients, we did not receive an answer. The majority of patients reported to be satisfied. Twelve out of twenty-nine (41%) patients were “very satisfied”, 6/29 (21%) were “satisfied”, 3/29 (10%) were “fairly satisfied, 1/29 (4%) was “fairly unsatisfied” and 2/29 (7%) were “very unsatisfied”. Overall, 21/29 (72%) patients were at least fairly satisfied about UAE, in 10/14 (71%) patients with pure adenomyosis and in 11/15 (73%) in patients with adenomyosis and fibroids (*p* = 0.552).

Twenty of 28 patients (71%) would advise UAE to a friend. Three would not (11%) and one (4%) patient responded not to know. Twenty patients would again undergo UAE, three not and one did not know. There were no differences between groups as detailed in Table 3.

**Table 3:** Satisfaction outcomes per group

	Pure adenomyosis (*n* = 11)	Adenomyosis with fibroids (*n* = 13)	*p* value
Very satisfied	5 (35.7%)	7 (46.7%)	0.552
Satisfied	2 (14.3%)	4 (26.7%)	
Fairly satisfied	3 (21.4%)	–	
Fairly unsatisfied	–	1 (6.7%)	
Very unsatisfied	1 (7.1%)	1 (6.7%)	
Advise a friend	10 (71.4%)	10 (66.7%)	0.348
Repeat embolization	10 (71.4%)	10 (66.7%)	

## Discussion

### Most Important Clinical Findings and Interpretations of Outcomes

Four of five patients had hysterectomy after the 37-month follow-up interval. The continued increase in patients undergoing hysterectomy underlines the importance of long-term follow-up. It could provide insights for prediction of quality of life, recurrence of symptoms, costs and possibly counseling of patients. The literature describes some varied secondary intervention and symptom improvement rates. Bae et al. followed up 50 patients with pure adenomyosis until 48 months after UAE and reported only one hysterectomy (at 18 months) and symptom improvement in 38/50 (76%) patients; however, this was not measured with a validated questionnaire. The secondary hysterectomy rate was not comparable to our study and could possibly be explained by; (1) the selected group of patients in our study with possibly worse baseline symptoms and no other option than a hysterectomy and 2) a longer follow-up than any other study in which we demonstrate that even at 55 and 80 months secondary hysterectomies are performed. Froeling et al. [16], that also used the UFS-QOL to measure HRQOL, reported a 2/7 hysterectomy rate in patients with pure adenomyosis and 2/10 hysterectomies rate in patients with adenomyosis and fibroids at a follow-up of 46 months. HRQOL scores were comparable to our study with a 44.0 score at baseline and 99.57 at 46 months of follow-up.

The most noticeable improvement of HRQOL in patients with pure adenomyosis and in adenomyosis with fibroids occurred in the first 3 months after UAE and remained stable over time without differences between the two groups. SSS at baseline was significantly different in favor of the adenomyosis combined with fibroids group. At follow-up, it showed comparable stable improvement at 3 and 37 months, but again displayed a small statistical difference in favor of patients with adenomyosis with fibroids at 7 years. This finding is concordant with a recent meta-analysis [12], but could also be explained by the initial selection bias were patients with pure adenomyosis had worse symptoms compared to the patients with adenomyosis and fibroids.

The majority of patients (72%) declared to be at least fairly satisfied about UAE, however one-third of patients was either not satisfied or indecisive. Menopause was reported by 42% of patients and did not reveal any differences between patients with adenomyosis and patient with adenomyosis and fibroids. We recognized that the age and subsequently menopause could bias the result in terms of high HRQOL and decreased SSS. Improvement not as result of UAE, but as a result of menopause and the absence of menstrual cycle related symptoms. However, since these women at baseline had no other treatment option than a hysterectomy when conservative management failed, a hysterectomy could be avoided in many of these patients. Therefore, we believe that embolization at the very least postponed the recurrence of symptoms and avoided major surgery in many women. The median age of our population was 50 (range 36–59). In developed countries, the average age of women for reaching menopause is 51 years [17].

### Strengths and Limitations

This is the longest follow-up cohort available so far. Other articles have reported long-term follow-up at 37, 40, 46, 48 and 58.8 months [7, 9, 13, 16, 18]. The cohort is relatively small which makes strong conclusions hazardous.

As reported earlier [15], there was no difference between patients with pure adenomyosis and patients with adenomyosis and fibroids. Unfortunately, these groups are heterogeneous in terms of adenomyosis and fibroids size, location and dominance; therefore, strong conclusions about what type of adenomyosis responds best to UAE may not be drawn.

## Conclusion

We conclude that after 7 years of follow-up, in 82% of patients UAE results in preservation of the uterus. Total of 72% of patients are at least fairly satisfied and 74% seem to respond well to UAE in terms of improvement of HRQOL and SSS.
